# Supplementary material for: EnzML: multi-label prediction of enzyme classes using InterPro signatures
Source: BMC Bioinformatics. 2012 Apr 25;13:61. doi: 10.1186/1471-2105-13-61 (PMC3483700; doi:10.1186/1471-2105-13-61)
Supplement: Addtional file 5 — The Java code to format the data files, evaluate and predict. The file enzml_java_code.tar.gz contains the Java code used to format database data to ARFF and XML formats, to execute cross and train-test (jackknife) evaluations and to record evaluation results to database. More information is included in the readme.txt file and the Javadoc files. The code can be used with a MySQL database. To use a different database software, other JDBC drivers might be required. [file 1471-2105-13-61-S5.gz › java_code/enzml2011/doc/uk/ac/ed/inf/csb/taweka/test/DataSetWriterTest.html]

DataSetWriterTest


---


|  |  |  |
| --- | --- | --- |
| **Overview** \| **Package** \| **Class** \| **Use** \| **Tree** \| **Deprecated** \| **Index** \| **Help** | |  |
| **PREV CLASS**   **NEXT CLASS** | **FRAMES**    **NO FRAMES**     **All Classes** |  |
| SUMMARY: NESTED \| FIELD \| CONSTR \| METHOD | DETAIL: FIELD \| CONSTR \| METHOD |  |


---


## uk.ac.ed.inf.csb.taweka.test Class DataSetWriterTest

```
java.lang.Object
  junit.framework.Assert
      junit.framework.TestCase
          uk.ac.ed.inf.csb.taweka.test.DataSetWriterTest
```

**All Implemented Interfaces:**: junit.framework.Test

---

``` public class DataSetWriterTest extends junit.framework.TestCase ```

Class

**Version:**
:   20 Feb 2008

**Author:**
:   Luna De Ferrari luna.deferrari-at-ed.ac.uk

---

| **Field Summary** | |
| --- | --- |
| `static java.lang.String` | `arffInstance1` |
| `static java.lang.String` | `arffInstance1b` |
| `static java.lang.String` | `arffInstance2` |
| `static java.lang.String` | `arffInstance4` |
| `static java.lang.String` | `ATT1` |
| `static java.lang.String` | `ATT1_TO_STRING` |
| `static java.lang.String` | `ATT2` |
| `static java.lang.String` | `ATT3` |
| `static java.lang.String` | `ATT3_TO_STRING` |
| `static java.lang.String` | `ATT4` |
| `static java.lang.String` | `ATT4_TO_STRING` |
| `static java.lang.String` | `CLASS_TO_STRING` |
| `static java.lang.String` | `CLASS1` |
| `static java.lang.String` | `CLASS2` |
| `static java.lang.String` | `CLASS3` |
| `static java.lang.String` | `INST1` |
| `static java.lang.String` | `INST2` |
| `static java.lang.String` | `INST3` |
| `static java.lang.String` | `INST4` |
| `static java.lang.String` | `m_arff` |
| `static java.lang.String` | `NAME` |


**Constructor Summary**

`DataSetWriterTest()`


| **Method Summary** | |
| --- | --- |
| `static java.util.Vector<java.lang.String>` | `attributes()`             Data: ATT1 ATT2 ATT3 CLASS instA y n n 1 instB y y n 2 instC n n y 2 |
| `static java.util.Vector<java.lang.String>` | `classValues()` |
| `static DataSetWriter` | `dataSet()` |
| `static java.lang.String` | `getExpectedArff()` |
| `static java.util.Vector<java.lang.String>` | `instances()` |
| `void` | `testGetArff()` |
| `void` | `testGetAttributes()` |
| `void` | `testGetName()`             public void testSetClass() { DataSetFactory data = DataSetFactoryTest.dataSet(); Enumeration instances = data.getDataSet().enumerateInstances(); Instance instA = (SparseInstance) instances.nextElement(); instA.setClassValue(DataSetFactoryTest.CLASS1); assertEquals("y", instA.stringValue(0)); assertEquals("y", instA.stringValue(1)); assertEquals("n", instA.stringValue(2)); assertEquals(DataSetFactoryTest.CLASS1, instA.stringValue(4)); assertEquals(DataSetFactoryTest.CLASS1, instA.stringValue(instA .classAttribute())); Instance instB = (SparseInstance) instances.nextElement(); assertEquals("n", instB.stringValue(0)); // assertEquals("n", instB.stringValue(1)); // assertEquals("y", instB.stringValue(2)); // assertEquals(DataSetFactoryTest.CLASS3, instB.stringValue(4)); // value1 assertEquals(DataSetFactoryTest.CLASS3, instB.stringValue(instB .classAttribute())); // value1 Instance instC = (SparseInstance) instances.nextElement(); assertEquals("n", instC.stringValue(0)); // y assertEquals("n", instC.stringValue(1)); // n assertEquals("n", instC.stringValue(2)); // n assertEquals(DataSetFactoryTest.CLASS2, instC.stringValue(4)); // value1 assertEquals(DataSetFactoryTest.CLASS2, instC.stringValue(instC .classAttribute())); } |
| `void` | `testLog()`             public void testCreateDataSet() { DataSetFactory dataSet = DataSetFactoryTest.dataSet(); Instances instances = dataSet.generateDataSet(); assertEquals(DataSetFactoryTest.NAME, instances.relationName()); } |

**Methods inherited from class junit.framework.TestCase**

`countTestCases, getName, run, run, runBare, setName, toString`

**Methods inherited from class junit.framework.Assert**

`assertEquals, assertEquals, assertEquals, assertEquals, assertEquals, assertEquals, assertEquals, assertEquals, assertEquals, assertEquals, assertEquals, assertEquals, assertEquals, assertEquals, assertEquals, assertEquals, assertEquals, assertEquals, assertEquals, assertEquals, assertFalse, assertFalse, assertNotNull, assertNotNull, assertNotSame, assertNotSame, assertNull, assertNull, assertSame, assertSame, assertTrue, assertTrue, fail, fail, failNotEquals, failNotSame, failSame, format`

**Methods inherited from class java.lang.Object**

`equals, getClass, hashCode, notify, notifyAll, wait, wait, wait`

**Field Detail**

### arffInstance1

```
public static final java.lang.String arffInstance1
```

**See Also:**: Constant Field Values

---


### arffInstance1b

```
public static final java.lang.String arffInstance1b
```

**See Also:**: Constant Field Values

---


### arffInstance2

```
public static final java.lang.String arffInstance2
```

**See Also:**: Constant Field Values

---


### arffInstance4

```
public static final java.lang.String arffInstance4
```

**See Also:**: Constant Field Values

---


### ATT1

```
public static final java.lang.String ATT1
```

**See Also:**: Constant Field Values

---


### ATT1\_TO\_STRING

```
public static final java.lang.String ATT1_TO_STRING
```

**See Also:**: Constant Field Values

---


### ATT2

```
public static final java.lang.String ATT2
```

**See Also:**: Constant Field Values

---


### ATT3

```
public static final java.lang.String ATT3
```

**See Also:**: Constant Field Values

---


### ATT3\_TO\_STRING

```
public static final java.lang.String ATT3_TO_STRING
```

**See Also:**: Constant Field Values

---


### ATT4

```
public static final java.lang.String ATT4
```

**See Also:**: Constant Field Values

---


### ATT4\_TO\_STRING

```
public static final java.lang.String ATT4_TO_STRING
```

**See Also:**: Constant Field Values

---


### CLASS\_TO\_STRING

```
public static final java.lang.String CLASS_TO_STRING
```

**See Also:**: Constant Field Values

---


### CLASS1

```
public static final java.lang.String CLASS1
```

**See Also:**: Constant Field Values

---


### CLASS2

```
public static final java.lang.String CLASS2
```

**See Also:**: Constant Field Values

---


### CLASS3

```
public static final java.lang.String CLASS3
```

**See Also:**: Constant Field Values

---


### INST1

```
public static final java.lang.String INST1
```

**See Also:**: Constant Field Values

---


### INST2

```
public static final java.lang.String INST2
```

**See Also:**: Constant Field Values

---


### INST3

```
public static final java.lang.String INST3
```

**See Also:**: Constant Field Values

---


### INST4

```
public static final java.lang.String INST4
```

**See Also:**: Constant Field Values

---


### m\_arff

```
public static java.lang.String m_arff
```

---


### NAME

```
public static final java.lang.String NAME
```

**See Also:**: Constant Field Values


**Constructor Detail**

### DataSetWriterTest

```
public DataSetWriterTest()
```


**Method Detail**

### testGetArff

```
public void testGetArff()
```

---


### testGetAttributes

```
public void testGetAttributes()
```

---


### testGetName

```
public void testGetName()
```

:   public void testSetClass() { DataSetFactory data =
    DataSetFactoryTest.dataSet(); Enumeration instances =
    data.getDataSet().enumerateInstances(); Instance instA = (SparseInstance)
    instances.nextElement(); instA.setClassValue(DataSetFactoryTest.CLASS1);
    assertEquals("y", instA.stringValue(0)); assertEquals("y",
    instA.stringValue(1)); assertEquals("n", instA.stringValue(2));
    assertEquals(DataSetFactoryTest.CLASS1, instA.stringValue(4));
    assertEquals(DataSetFactoryTest.CLASS1, instA.stringValue(instA
    .classAttribute()));
    Instance instB = (SparseInstance) instances.nextElement();
    assertEquals("n", instB.stringValue(0)); // assertEquals("n",
    instB.stringValue(1)); // assertEquals("y", instB.stringValue(2)); //
    assertEquals(DataSetFactoryTest.CLASS3, instB.stringValue(4)); // value1
    assertEquals(DataSetFactoryTest.CLASS3, instB.stringValue(instB
    .classAttribute())); // value1
    Instance instC = (SparseInstance) instances.nextElement();
    assertEquals("n", instC.stringValue(0)); // y assertEquals("n",
    instC.stringValue(1)); // n assertEquals("n", instC.stringValue(2)); // n
    assertEquals(DataSetFactoryTest.CLASS2, instC.stringValue(4)); // value1
    assertEquals(DataSetFactoryTest.CLASS2, instC.stringValue(instC
    .classAttribute()));
    }

---


### testLog

```
public void testLog()
```

:   public void testCreateDataSet() { DataSetFactory dataSet =
    DataSetFactoryTest.dataSet(); Instances instances =
    dataSet.generateDataSet(); assertEquals(DataSetFactoryTest.NAME,
    instances.relationName()); }

---


### attributes

```
public static java.util.Vector<java.lang.String> attributes()
```

:   Data: ATT1 ATT2 ATT3 CLASS instA y n n 1 instB y y n 2 instC n n y 2

---


### classValues

```
public static java.util.Vector<java.lang.String> classValues()
```

---


### dataSet

```
public static DataSetWriter dataSet()
```

---


### getExpectedArff

```
public static java.lang.String getExpectedArff()
```

---


### instances

```
public static java.util.Vector<java.lang.String> instances()
```


---


|  |  |  |
| --- | --- | --- |
| **Overview** \| **Package** \| **Class** \| **Use** \| **Tree** \| **Deprecated** \| **Index** \| **Help** | |  |
| **PREV CLASS**   **NEXT CLASS** | **FRAMES**    **NO FRAMES**     **All Classes** |  |
| SUMMARY: NESTED \| FIELD \| CONSTR \| METHOD | DETAIL: FIELD \| CONSTR \| METHOD |  |


---
